# Structural and Computational Insights into Transketolase-like 1 (TKTL-1): Distinction from TKT and Implications for Cancer Metabolism and Therapeutic Targeting

**DOI:** 10.3390/molecules30193905

**Published:** 2025-09-27

**Authors:** Ahmad Junaid, Caleb J. Nwaogwugwu, Sameh H. Abdelwahed

**Affiliations:** Department of Chemistry, Prairie View A&M University, Prairie View, TX 77446, USA

**Keywords:** TKTL-1, cancer metabolism, transketolase, pentose phosphate pathway, therapeutic targeting, computational analysis, structure-based drug design, MMPBSA analysis, selective inhibitors

## Abstract

Transketolase-like protein 1 (TKTL-1) has been implicated in altered cancer metabolism, yet its structure and molecular function remain poorly understood. In this study, we established a homology model of TKTL-1 using multiple templates and validated it through sequence alignment and structural comparison with the canonical transketolase (TKT). Binding-site identification was performed using CASTp, receptor cavity mapping, and blind docking, all of which consistently pointed to a conserved region involving interactive residues shared between TKT and TKTL-1. Comparative docking revealed the reduced affinity of TKTL-1 for TDP, supporting functional divergence between TKTL-1 and TKT. We further analyzed conserved residues and receptor surfaces, which enabled us to propose predictive scaffolds as potential modulators of TKTL-1. While these scaffolds remain theoretical, they provide a computational framework to guide future pharmacophore modeling, molecular dynamics simulations, and experimental validation. Together, our study highlights the structural features of TKTL-1, establishes its key differences from TKT, and lays the groundwork for future drug discovery efforts targeting cancer metabolism.

## 1. Introduction

The pentose phosphate pathway (PPP) is a central metabolic route that supports both biosynthetic and redox homeostasis. It provides ribose-5-phosphate (R-5P) for nucleotide synthesis and generates nicotinamide adenine dinucleotide phosphate (NADPH), a critical reducing agent for anabolic reactions and cellular defense against oxidative stress. The pathway is divided into two phases: an oxidative phase that produces NADPH and R-5P, and a non-oxidative phase that interconverts sugars to meet cellular demands [1,2,3,4,5,6].

**Figure 1:**
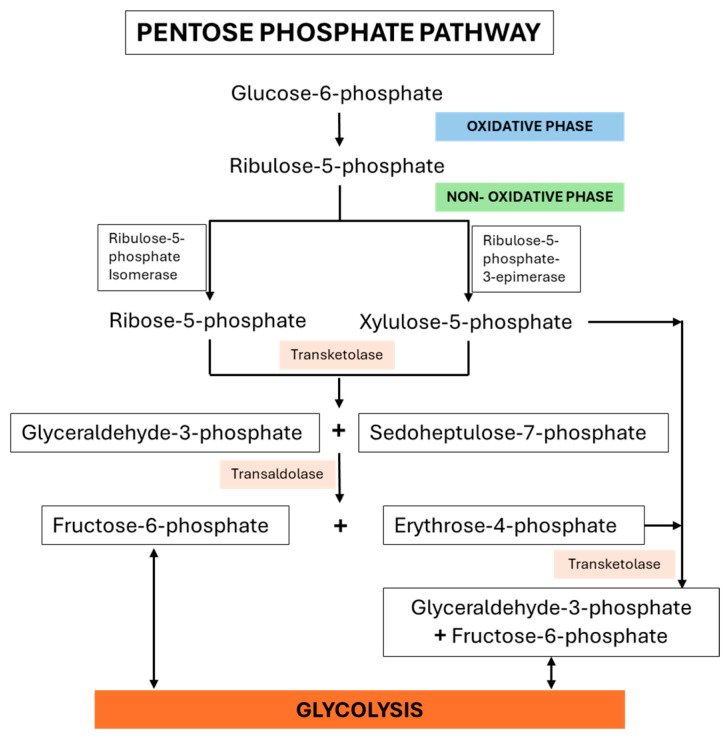
Transketolase (TKT)-mediated non-oxidative branch of the pentose phosphate pathway (PPP).

In the oxidative phase, glucose-6-phosphate is oxidized to yield ribulose-5-phosphate (Ru-5P), alongside the reduction in two molecules of NADP^+^ to NADPH [7,8]. During the non-oxidative phase, transketolase (TKT), a thiamine diphosphate-dependent enzyme, catalyzes the transfer of two-carbon units between sugar phosphates. This activity converts R-5P into glyceraldehyde-3-phosphate (G-3P) and sedoheptulose-7-phosphate (S-7P) and, in subsequent reactions with transaldolase (TALDO), generates erythrose-4-phosphate (E-4P) and fructose-6-phosphate (F-6P) [6,9,10]. A secondary TKT reaction further interconverts E-4P and xylulose-5-phosphate (X-5P) into F-6P and G-3P. While G-3P can enter glycolysis, F-6P may cycle back into the oxidative phase, reinforcing NADPH production (Figure 1).

TKT-mediated control of the non-oxidative PPP is particularly relevant in rapidly proliferating cells, such as cancer cells, where nucleotide and NADPH demands are elevated [11,12,13,14,15,16]. Indeed, it has been estimated that nearly 80% of ribonucleotides in cancer cells derive from this branch of the PPP [17]. In this context, TKT exhibits metabolic flexibility, including reverse activity when the demand for nucleotides exceeds that for NADPH, highlighting its adaptive role in cellular metabolism.

In humans, the *TKT* gene coexists with two homologous genes, *TKTL-1*, and *TKTL-2* [18,19,20]. *TKTL-1* exhibits 66% similarity to *TKT* at the DNA level and 63% at the protein level, while *TKTL-2* demonstrates a higher similarity, with 80% on the DNA level and 77% on the protein level. In 2005, the TKTL-1 protein was identified in human tumor cells, initially presumed to be a mutant form of hTKT [21]. Although it was previously suggested to possess transketolase (TKT) activity, direct experimental evidence confirming this enzymatic activity in TKTL-1 has not been reported. Furthermore, TKTL-1 differs from TKT by the absence of 38 amino acids. Immunohistochemical studies have revealed the significance of these missing amino acids in TKTL-1, as they play a crucial role in anchoring the Thiamin diphosphate (TDP) co-factor and facilitating catalytic activity [22]. Importantly, TKTL-1 is frequently overexpressed in cancers—including colon, urothelial, gastric, and lung tumors—and its upregulation correlates with poor prognosis and chemoresistance [23,24,25,26,27].

### 1.1. Distinctive Characteristics of TKT and TKTL-1 in Cellular Metabolism

The functional role of transketolase-like 1 (TKTL-1) has been the subject of considerable debate. While some reports suggested that TKTL-1 exhibits transketolase (TKT) activity, direct experimental evidence is lacking. Early work by Coy et al. reported TKT activity in recombinant TKTL-1; however, the study lacked specific details on protein preparation and general characteristics [21]. Another study investigated the computational basis for the similarity between TKT and TKTL-1 [28]. Although homology modeling suggested potential catalytic activity in TKTL-1, the key amino acids responsible for Thiamin diphosphate (TDP) binding were reportedly absent, and the replacement amino acids were not identified [28]. Experimental studies using the TKTΔ38 variant, which mirrors the 38 amino acid deletion characteristics of TKTL-1, confirmed the loss of enzymatic activity and inability to bind TDP [22]. Similarly, recombinant TKTL-1 protein failed to interact with TDP [29], providing strong evidence that TKTL-1 does not function as a canonical transketolase. The proposed role of TKTL-1 as a target of the transketolase inhibitor oxythiamin (OT) has also been refuted, as the absence of critical residues such as His102 precludes binding to TDP or OT [30,31]. This lack of affinity to TDP was further confirmed when recombinant TKTL-1 exhibited a lack of binding to TDP [29].

Despite lacking proven transketolase activity, TKTL-1 plays a distinctive role in cancer biology. Expression profiling across various tumor types demonstrates that TKTL-1 is selectively upregulated, whereas TKT and TKTL-2 expression remain unchanged [23,24]. This selective induction suggests that cancer cells specifically exploit TKTL-1 to support growth and survival. In contrast, TKT remains a well-characterized enzyme central to the non-oxidative pentose phosphate pathway and glycolysis. Together, these findings underscore that TKTL-1, while structurally related to TKT, is functionally divergent, contributing to metabolic reprogramming and tumor progression.

### 1.2. TKTL-1 in Cancer Progression and Metabolic Adaptation

**Figure 2:**
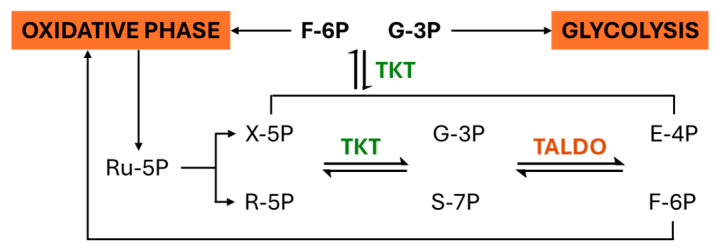
TKT-mediated feedback mechanism and hypoxic adaptation in TKTL1-driven cancer progression.

Altered metabolism is a hallmark of cancer, with the Warburg effect, preferential use of glycolysis for energy production even in the presence of oxygen, being a defining feature. This shift supports the accumulation of biosynthetic precursors required for rapid cell growth. TKTL-1 has been implicated in this process by driving the non-oxidative branch of the pentose phosphate pathway (PPP), where ribose-5-phosphate (R-5P) serves as a precursor for nucleotide synthesis and NADPH supports lipid synthesis and redox balance (Figure 2). Elevated TKTL-1 expression has been documented across several cancer types, correlating with enhanced proliferation, invasion, metastasis, and resistance to apoptosis. Functional studies in HeLa cells revealed that TKTL-1 overexpression increases R-5P levels twofold, whereas TKTL-1 knockdown reduces R-5P by 35–40% [29]. Moreover, TKTL-1 and R-5P levels peak in the G1/S phase, aligning with nucleotide demand for DNA replication. These findings suggest that TKTL-1 contributes to the Warburg effect by sustaining nucleotide availability, accounting for up to 85% of RNA sugars in cancer cells [32].

Mechanistically, TKTL-1 appears tightly regulated by cadherin-1 (*CDH-1*), a tumor suppressor gene. *CDH-1* directly binds TKTL-1 via a destruction box (D-box) motif (RXXLXXXD/N/E; TKTL-1 sequence: RGTLQVLQD), which is absent in canonical TKT [33]. In normal cells, *CDH-1* restrains TKTL-1 levels, whereas *CDH-1* downregulation in cancer leads to unchecked TKTL-1 accumulation. Mutant TKTL-1 lacking the D-box fails to interact with *CDH-1*, becoming resistant to this regulatory control [29]. Functionally, this CDH-1–TKTL-1 axis directly influences DNA synthesis: *CDH-1* knockdown increases DNA synthesis in a TKTL-1–dependent manner, while TKTL-1 overexpression enhances DNA replication as measured by EdU incorporation [29]. Together, these observations highlight TKTL-1 as a driver of metabolic adaptation in cancer cells and reveal a critical regulatory pathway linking *CDH-1* loss to uncontrolled nucleotide biosynthesis. Targeting this interaction may therefore represent a promising therapeutic strategy to starve tumor cells of essential metabolic intermediates.

**Figure 3:**
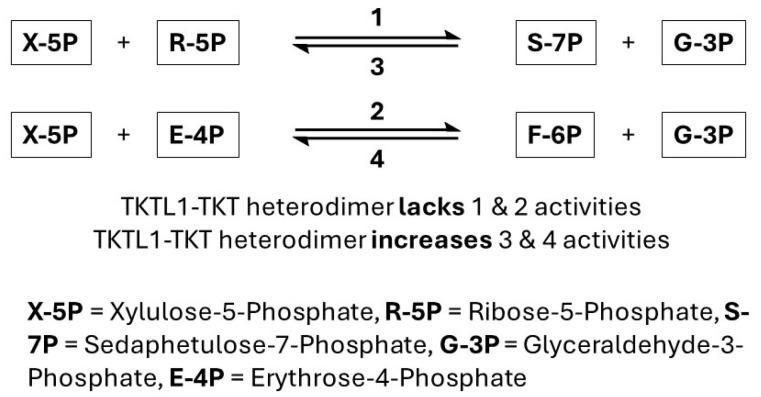
Four Transketolase activities.

Further, tandem affinity purification revealed that TKTL-1 forms heterodimers with TKT monomers, with affinity comparable to TKT homodimers [29]. Functional assays showed that while TKT homodimers retain all canonical activities, TKTL-1–TKT heterodimers selectively enhance reverse PPP reactions while reducing forward activities. The absence of His103 in TKTL-1 likely contributes to this shift by altering TDP binding and substrate specificity (Figure 3) [30,31,34]. This bias toward reverse activity may allow cancer cells to sustain nucleotide synthesis under aerobic conditions, reinforcing the Warburg effect. Consistently, pancreatic cancer cells have been shown to increase nucleic acid synthesis via enhanced non-oxidative PPP flux, with a preference for fructose over glucose substrates [35].

### 1.3. Therapeutic Potential of Targeting TKT-1

The consistent overexpression of TKTL-1 in diverse cancers highlights it as a promising therapeutic target. Beyond oncology, TKTL-1 has also been implicated in neurodegenerative disorders, including Wernicke–Korsakoff syndrome [21,36], Alzheimer’s disease [21], and diabetic neuropathy associated with diabetes mellitus [21,37]. Given its potential roles across multiple disease contexts, TKTL-1 should be regarded as distinct from classical TKT and investigated accordingly. The development of selective TKTL-1 modulators would provide critical tools to dissect its precise function. For example, inhibitors could be employed to quantify the contribution of the non-oxidative PPP to nucleotide synthesis or tested in combination with TKT inhibitors to evaluate possible synergistic anticancer effects. By restricting nucleotide and sugar availability, TKTL-1 inhibitors may specifically impair cancer cell proliferation while minimizing off-target effects associated with conventional antiproliferative drugs. Conversely, TKTL-1 activators could serve as valuable probes to clarify its role in neurodegenerative conditions and potentially mitigate disease progression.

## 2. Results

**Figure 4:**
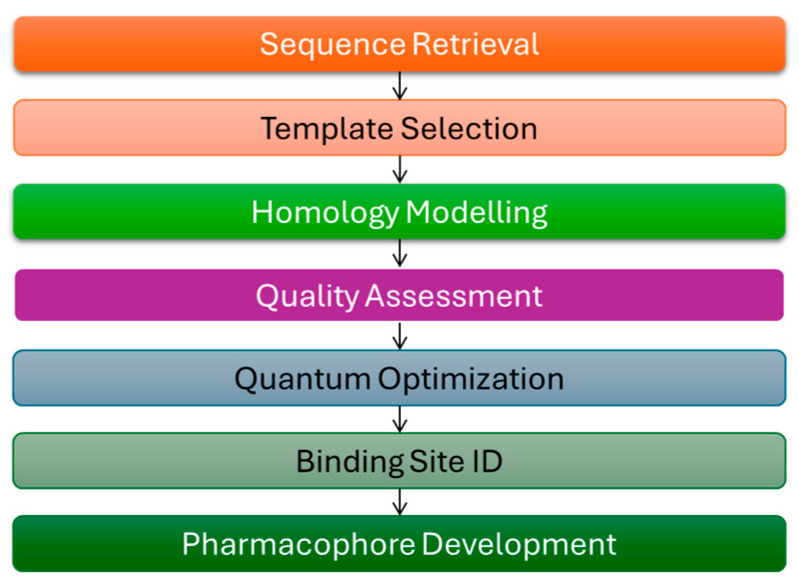
Schematic of computational workflow.

The computational workflow applied in this study is outlined in Figure 4. Starting with sequence retrieval, we progressed through multi-template selection, homology model construction, and stereochemical validation, followed by docking and binding energy calculations. Together, these steps provided a clear and reproducible path for building the TKTL-1 structural model and examining its functional divergence from TKT.

### 2.1. Homology Model Generation of TKTL-1

The initial crystallization trial for isolating TKTL-1 was not successful [22], and, to date, no experimentally resolved structure is available. TKTL-1 has specific insertions and substitutions as compared to TKT that may affect its folding and stability, potentially leading to misfolding, aggregation or reduced expression [22]. These structural features could explain the reported changes in obtaining a stable protein crystal structure. Therefore, a multi-template homology modeling strategy was adopted to generate a reliable 3D model of TKTL-1. The full-length human TKTL-1 amino acid sequence (UniProt ID: P51854) was retrieved and aligned to five structurally related templates (Table 1) using the Modeller tool in Discovery Studio [38]. The best model with the lowest Z-score was selected for further studies (The Z-score indicates the overall quality of the protein model, with more negative values reflecting better and more reliable structures.).

**Figure 5:**
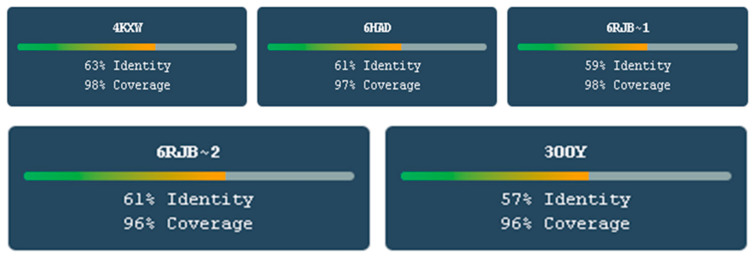
Comparative sequence identity analysis of five crystal structure templates used for TKTL-1 homology modeling.

The comparative analysis of five crystal structure templates used for TKTL-1 homology modeling is shown in Figure 5, with sequence identity ranging from 57 to 63% and structural coverage of 96–98%. The gradient bars visually represent the quality spectrum, with 4KXW showing the highest sequence identity (63%) and coverage (98%), making it the optimal template for model generation. The consistently high coverage across all templates (>96%) ensures comprehensive structural representation of TKTL-1, while the sequence identity values above 57% exceed the threshold required for reliable homology modeling. This multi-template approach validates the robustness of the final TKTL-1 model by demonstrating that multiple high-quality structural templates support the same overall fold, providing confidence in the subsequent binding studies and drug design applications that depend on accurate 3D structure representation.

### 2.2. Structural Validation of the Homology Model

After the generation of homology modeling, RAMPAGE was used for the assessment of TKTL-1 homology models [39]. Ramachandran plots of the models were generated to assess the generated models.

**Figure 6:**
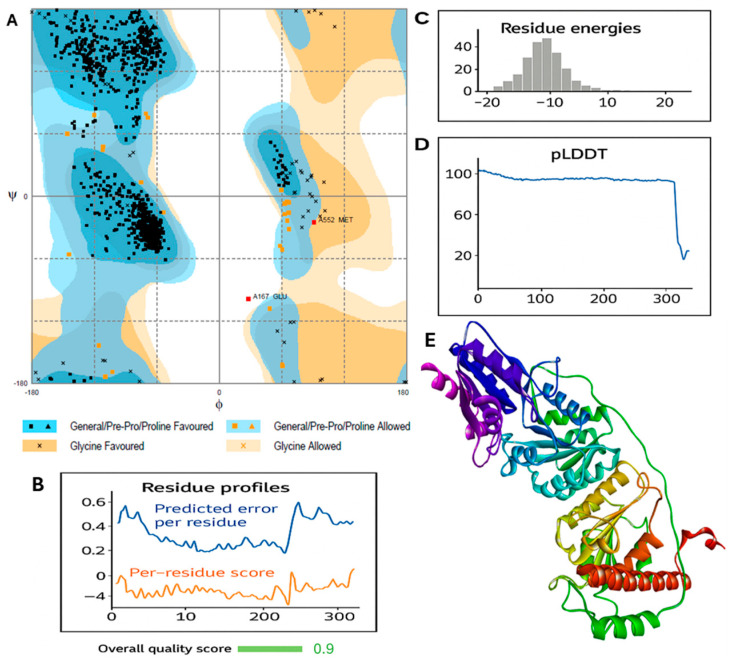
Model quality metrics: (**A**) Ramachandran Plot, dots in blue represents number of residues in favored region (~98.0% expected), dots in orange represents number of residues in allowed region (~2.0% expected), dots in white represents number of residues in outlier region: 2 (0.2%) (**B**) residue profile analysis (**C**) residue energy distribution, (**D**) predicted local distance difference test (pLDDT), (**E**) 3D ribbon representation.

Model validation using structural quality metrics (Figure 6, Table 2) showed that 98% of residues were in favored or allowed regions of the Ramachandran plot, with high pLDDT scores across most of the sequence and predominantly negative residue energies, confirming the accuracy and stability of the TKTL-1 homology model.

### 2.3. Functional Divergence of TKT and TKTL-1 in TDP Binding

**Figure 7:**
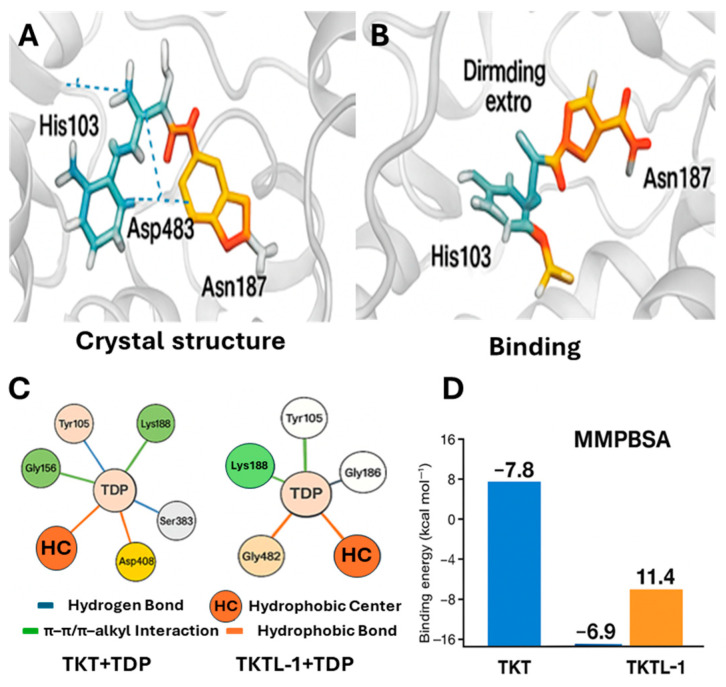
TDP-binding comparison between TKT and TKTL-1: (**A**) human TKT bound to thiamin diphosphate (TDP), (**B**) docked structure of TKTL-1 with TDP, (**C**) key interactions of TDP with TKT and TKTL-1, (**D**) binding energy of TDP with TKT and TKTL-1.

To validate the docking protocol, TDP was re-docked into human TKT (PDB ID: 4KXW). The docked pose reproduced the crystallographic binding mode with an RMSD < 0.3 Å, confirming methodological accuracy. The predicted binding energy was −7.8 kcal/mol with a dissociation constant of 2.0 × 10^6^ pM, and key hydrogen bonds were observed between TDP and His103, Asp483, and Asn187, consistent with literature reports (Figure 7A,C,D) [40].

In contrast, docking TDP into the TKTL-1 homology model revealed a marked loss of binding affinity (binding energy = −6.9 kcal/mol; Kd = 7.8 × 10^6^ pM) and an unfavorable binding free energy of +11.4 kJ/mol from MM-PBSA calculations (Figure 7B,D). Although His103 and Asn187 were conserved, the absence of Asp483—and its replacement by a misaligned or non-homologous residue likely disrupts cofactor coordination. This structural change directly explains TKTL-1’s reduced TDP affinity and supports the conclusion that TKTL-1 functions as a non-catalytic regulatory protein rather than an active transketolase enzyme.

### 2.4. Sequence and Structural Comparison of TKT and TKTL-1

**Figure 8:**
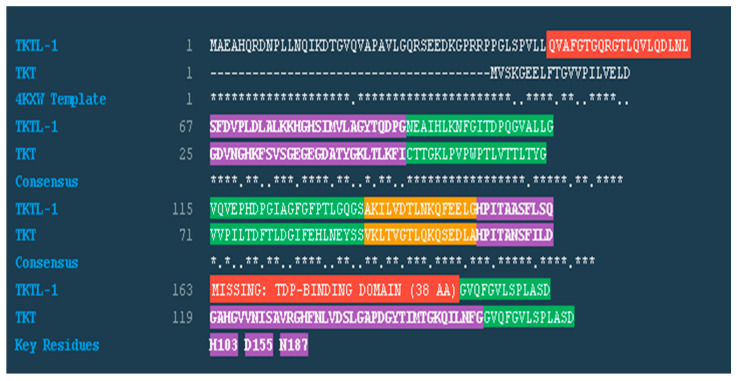
Multiple sequence alignment and 3D structural comparison. Green represents fully conserved region, denoted by (*), Yellow represents similar region, red represents 38 AA deletion in TKTL-1, purple represents TDP binding residues.

Multiple sequence alignment of TKT, TKTL-1, and the 4KXW template (Figure 8) revealed a defining 38-residue deletion in TKTL-1 that removes key thiamine diphosphate (TDP)–binding residues (His103, Asp155, Asn187) essential for cofactor coordination in TKT. Despite 67.8% overall sequence similarity, conservation mapping indicates that while the structural scaffold is largely preserved, critical catalytic motifs are absent.

The consensus pattern supports a model in which TKTL-1 retains sufficient structural compatibility to form heterodimers with TKT but lacks the enzymatic machinery for TDP binding. This is consistent with the positive binding free energy observed in MMPBSA calculations (+11.44 kJ/mol), suggesting a non-catalytic, regulatory role rather than independent transketolase activity.

**Figure 9:**
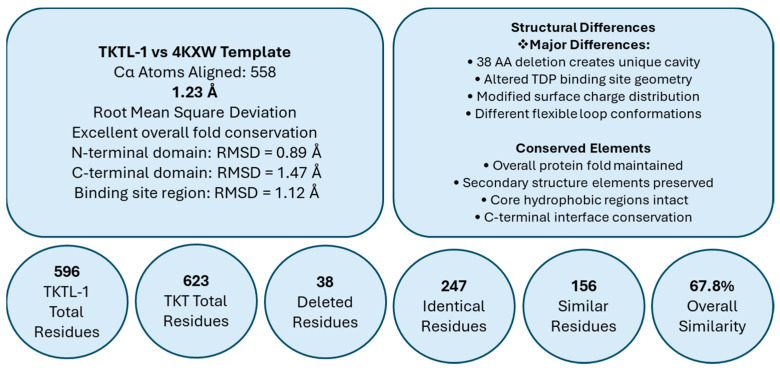
Structural conservation and divergence between TKTL-1 and the 4KXW template.

Homology modeling confirmed strong fold conservation with the 4KXW template (RMSD = 1.23 Å over 558 Cα atoms; Figure 9). Domain-level analysis showed higher structural preservation in the N-terminal domain (RMSD = 0.89 Å) and binding site region (RMSD = 1.12 Å) than in the more flexible C-terminal domain (RMSD = 1.47 Å). The deletion produces a unique cavity and altered TDP-binding geometry but leaves the global fold, secondary structure organization, and C-terminal dimerization interface intact.

Quantitatively, TKTL-1 contains 596 residues versus 623 in TKT, with 247 identical and 156 similar residues, confirming 67.8% sequence identity/similarity. Together, these data define TKTL-1 as a structurally conserved but catalytically impaired transketolase-like protein, supporting its classification as a regulatory modulator of TKT rather than a conventional enzyme.

### 2.5. Computational Optimization

To gain insights into the binding affinities and stability of interactions involving thiamin diphosphate (TDP), transketolase (TKT), and transketolase-like 1 (TKTL-1), we performed detailed computational optimization. This process involves using quantum chemical calculations to determine the electronic structures and lowest energy configurations of the individual molecules and their complexes.

#### 2.5.1. Computational Optimization of Thiamin Diphosphate (TDP)

TDP was geometry-optimized using the Full Optimization (FOPT) method with a Restricted Hartree–Fock (RHF) approach and a 6-31+G basis set. The system was modeled at physiological pH and a singlet spin state and solvation effects, using polarizable continuum model (PCM) for water, to attain the lowest energy configuration (Table 3).

**Figure 10:**
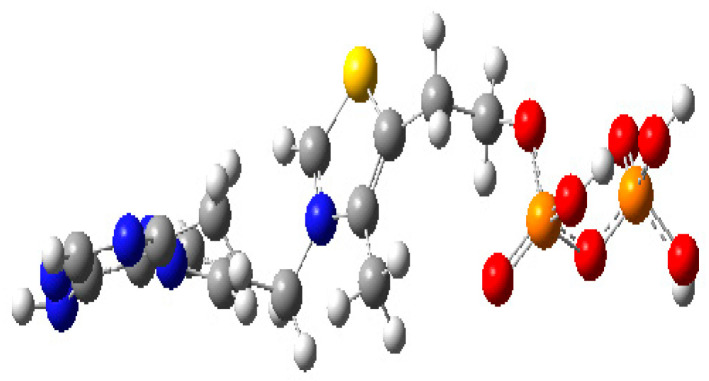
Thiamine diphosphate optimized structure. Blue and yellow represents nitrogen and sulfur atoms of thiamine, and red and orange color represents oxygen and phosphorous atoms of phosphate group.

The tight SCF (self-consistent field) convergence criterion of 8.0 × 10^−6^ Hartree/Bohr indicates successful convergence toward a minimum energy state. The optimized geometry (Figure 10) exhibited a low RMS gradient and no imaginary frequencies, confirming that the structure corresponds to a stable minimum-energy configuration.

#### 2.5.2. Computational Optimization of Transketolase (TKT)

TKT optimization was performed using FOPT with the Unrestricted Hartree–Fock (UHF) method and a 6-31+G basis set (Table 4). The open-shell doublet nature reflects its electronic configuration.

**Figure 11:**
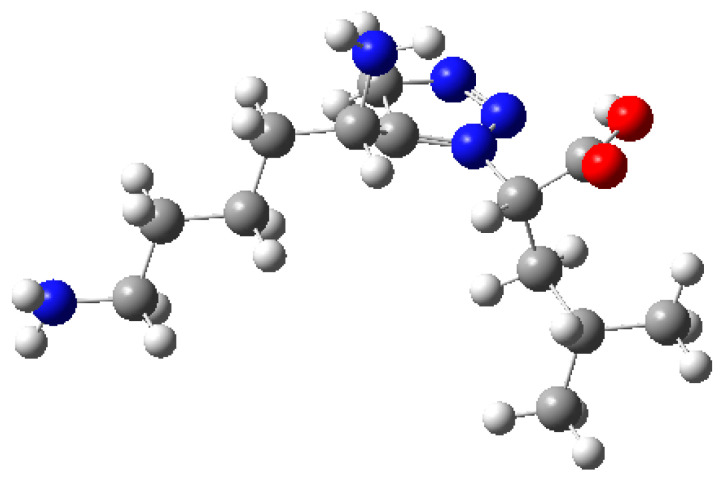
Transketolase (TKT)-optimized structure. Atoms are color-coded as follows: carbon (grey), hydrogen (white), nitrogen (blue), and oxygen (red).

The optimization converged with a low SCF convergence and the absence of imaginary vibrational frequencies, confirmed that the optimized structure represents a true minimum on the potential energy surface (Figure 11). The dipole moment of 6.580 Debye indicates moderate molecular polarity, consistent with the expected electronic distribution of TKT. Although the current calculation was performed in the gas phase without explicit solvation, the results provide a reliable baseline structure for subsequent docking and binding studies. Inclusion of solvent models and dynamic simulations will further refine TKT’s conformational and electronic properties.

#### 2.5.3. Computational Optimization of Transketolase-like 1 (TKTL-1)

**Figure 12:**
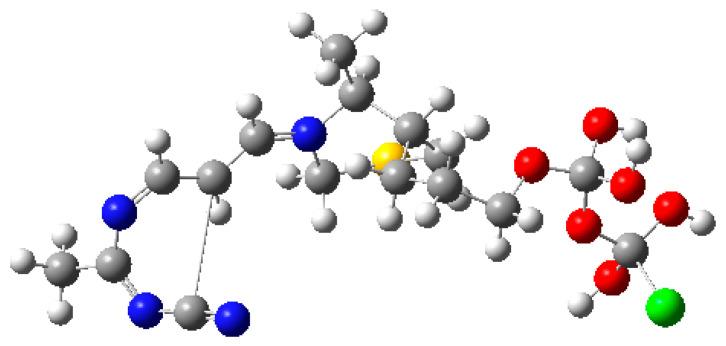
Optimized structure of Transketolase-Like 1 (TKTL-1). The molecular model corresponds to a TKTL-1 fragment, displaying its carbon backbone (grey), nitrogen-containing groups (blue), and polar oxygen atoms (red). The green sphere represents a chloride counterion included for charge neutrality in the quantum chemical model.

TKTL-1 was optimized using the same FOPT/RHF/6-31+G approach (Table 5). The optimized configuration (Figure 12) had a negative electronic energy and large dipole moment, indicating a stable yet highly polar structure.

The optimization converged successfully with a tight SCF convergence, and no imaginary frequencies, confirming that the obtained structure corresponds to a true minimum on the potential energy surface. The optimized geometry exhibits a dipole moment of 7.731 Debye, indicative of substantial molecular polarity and charge separation.

This polarity, combined with the stable optimized configuration, suggests that TKTL-1 may engage in biologically relevant electrostatic and hydrogen-bonding interactions. While the absence of an explicit solvation model limits the description of its behavior in aqueous environments, the current gas-phase optimization provides a stable and energetically favorable baseline structure. Nonetheless, these computational optimization results offer valuable insights into TKTL-1’s stability and electronic properties, forming a basis for further exploration of its biological functions and interactions.

#### 2.5.4. Computational Optimization of TKT and TDP Complex

**Figure 13:**
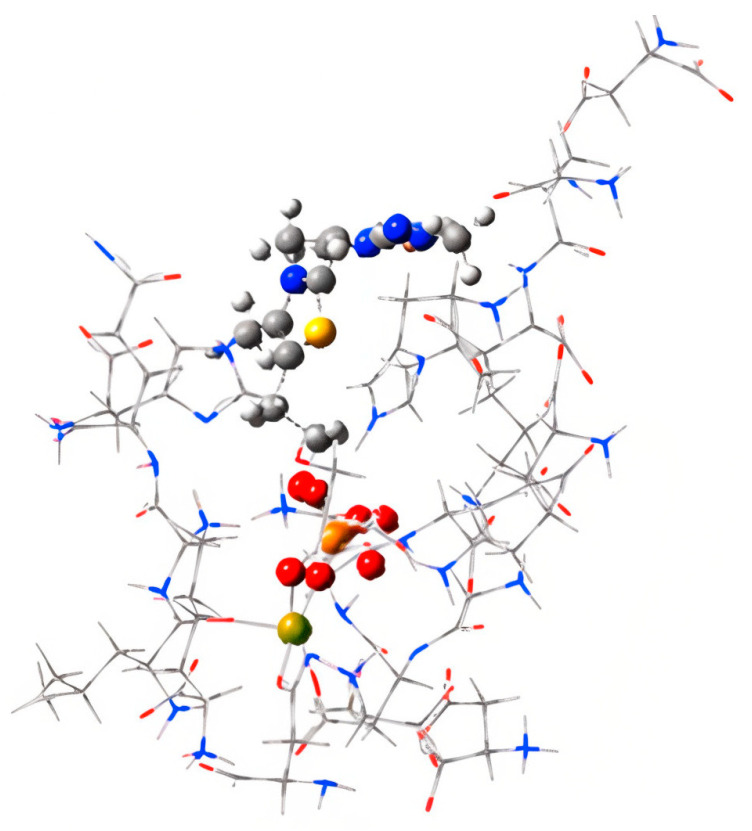
Optimized structure of the Transketolase-Like 1 (TKTL-1) fragment in complex with Thiamine Diphosphate (TDP). The thiamine diphosphate (TDP) cofactor is depicted in ball-and-stick representation (C, grey; N, blue; O, red; P, orange; S, yellow), coordinated with the catalytic Ca^2+^ ion (green sphere).

The TKT–TDP complex was optimized using FOPT UHF/6-31+G. The moderate dipole moment indicates balanced polarity (Table 6). The negative electronic energy represents the stable and energetically favorable molecular configuration. The dipole moment value of 4.8161 Debye indicates a moderate molecular polarity. Figure 13 displays a structural representation of the optimized TKT-TDP complex.

#### 2.5.5. Computational Optimization of TKTL-1 and TDP Complex

The TKTL-1–TDP complex was optimized using FOPT/RHF/6-31+G. The system, characterized by a neutral charge and a singlet spin state, was simulated without solvation effects (Table 7). The high dipole moment suggests significant polarity at the binding interface.

**Figure 14:**
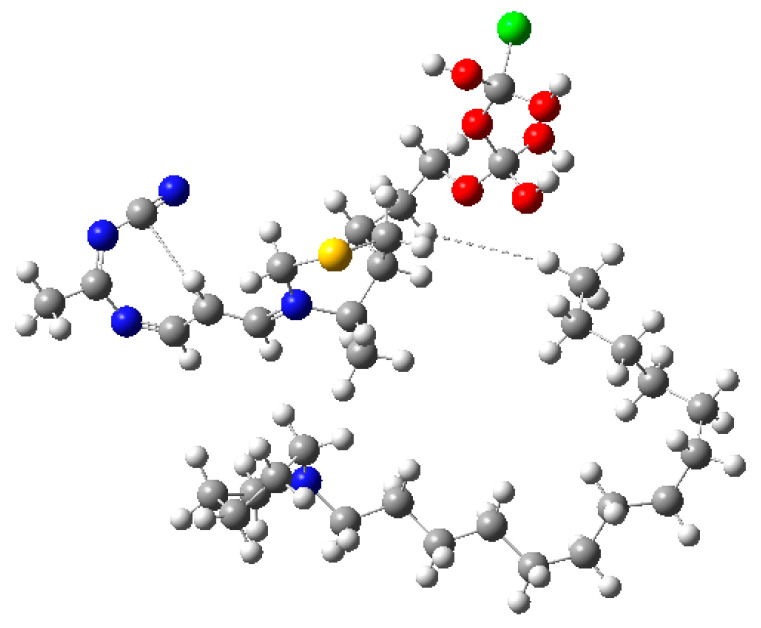
Optimized structure of the Transketolase-Like 1 (TKTL-1) fragment in complex with Thiamine Diphosphate (TDP). The TDP cofactor is visible with its 4-amino-2-methylpyrimidine ring (blue nitrogen), thiazolium sulfur (yellow), and diphosphate group (red oxygens). The green sphere represents a chloride counterion included for charge neutrality in the quantum chemical model.

The optimization converged successfully with no imaginary frequencies (Figure 14). The optimized dipole moment of 8.449 Debye indicates a highly polar interface between TKTL-1 and TDP, suggesting strong electrostatic interactions despite the generally weak binding affinity observed in MM-PBSA calculations (Figure 7D).

Table 8 presents a side-by-side comparison of the optimized structures for TDP, TKTL-1, TKT, and their complexes, with a focus on stability and polarity. In almost all cases, the calculations converged cleanly, with tight SCF convergence. This means the structures reached true minima on the potential energy surface, representing stable configurations.

While this computational optimization offers useful insight into relative stability, it does not replace a formal MM-PBSA calculation (see Section 2.3), which requires molecular dynamics (MD) trajectories and consistent force-field energies. The optimized TDP–TKT complex exhibited a stable, energetically favorable interaction. Structural convergence (no imaginary frequencies) and moderate polarity supported the formation of a strong binding interface, consistent with the well-established role of TKT as a thiamine diphosphate (TDP)–dependent enzyme. In contrast, the optimized TDP–TKTL-1 complex, although geometrically stable, displayed unfavorable binding characteristics. The absence of critical TDP-coordinating residues (e.g., Asp483, His103) and a 38-residue deletion disrupted cofactor binding (Figure 7D), leading to a weak and unstable interaction compared with the TDP–TKT complex.

Overall, these findings indicate that TKT forms a favorable and stable complex with TDP, in line with its enzymatic function, whereas TKTL-1 fails to establish stable binding. However, its distinct dipole moment suggests TKTL-1 may function as a separate enzyme, reinforcing the conclusion that TKTL-1 is structurally and functionally distinct from TKT.

### 2.6. Binding Site Identification

**Figure 15:**
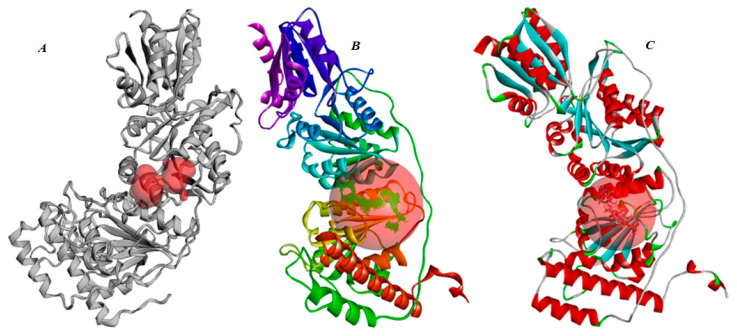
Binding site identification by (**A**) CASTp (**B**) receptor cavity via Discovery studio and (**C**) by blind docking of TKTL-1 homology model.

Given the markedly reduced TDP affinity observed for TKTL-1 at the equivalent TKT binding site, we performed a systematic binding site analysis of the TKTL-1 homology model. Binding site characterization is a critical prerequisite for structure-based drug design, particularly when targeting catalytically altered proteins. Three complementary approaches were employed: CASTp (Computer Atlas of Surface Topography of proteins [41] receptor cavity (via discovery studio) and blind docking of TKTL-1 enzyme techniques were utilized for bind site identification (Figure 15).

All three methods converged on the same surface pocket and identified an identical set of interactive residues: Phe61, Tyr67, Pro72, Gly73, Asn74, Asn77, Asp78, Arg79, Phe80, Val81, Phe88, Ala92, Thr93, Gly94, Trp95, Gln98, Gly99, Val102, Met106, Ile272, Thr273, Asp274, Val275, Arg308, Leu348, Ala351, Ser352, Arg353, Gly354, Thr356, Ile357, Ile376, Ala380, Glu381, Asn383, Ala436, Asn437, Lys439, and Gly440.

Of particular interest, residues Ala436, Asn437, Lys439, and Gly440—located near the C-terminal region—were consistently included in the binding site prediction across all protocols. This is notable given that TKTL-1 interacts with TKT to form a heterodimer via its C- or N-terminal region (amino acids 448–596). While removal of the terminal segment abolishes TKT binding, deletion of the central portion does not, suggesting the terminal region’s role in both protein–protein and ligand interactions.

**Figure 16:**
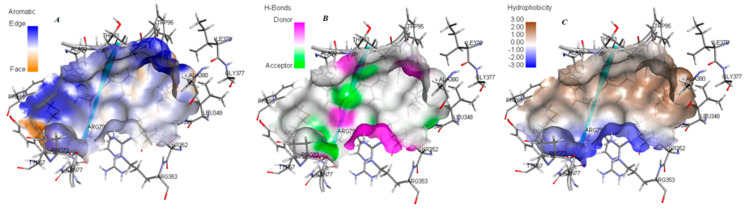
Receptor surfaces of the binding site based on (**A**) aromaticity, (**B**) H-bonding, (**C**) hydrophobicity.

Figure 16 visualize the amino acid composition and physicochemical properties of the identified site. The receptor surface analysis highlights hydrophobic patches, hydrogen-bond donors/acceptors, and aromatic interaction zones, offering key design cues for scaffold development. Collectively, these findings define a druggable pocket in TKTL-1 that is conserved across independent prediction methods, providing a promising target for selective inhibitor design.

### 2.7. Proposed TKTL-1 Modulator Scaffolds

Based on the comprehensive binding site analysis and surface property characterization, we propose three distinct classes of heterocyclic scaffolds specifically targeting TKTL-1, which will be the subject of future validation. The first class included quinazoline-based molecules designed to competitively engage in the identified pockets through π-π stacking interactions and hydrogen bonding with pocket residues. The second class comprises benzimidazole derivatives that may interfere with TKTL-1/TKT heterodimer formation and may provide dual inhibitory effect. The third class focuses on pyrimidine-based derivatives, including a pyrimidine-2-thiol scaffold, that targets the putative allosteric site unique to TKTL-1. These scaffolds are rationally designed to modulate the TKTL-1 activity by exploiting the physiochemical features of the proposed pocket. Detailed docking studies, MD simulations and experimental validations will be reported in the future studies.

## 3. Discussion

This study applied an integrated computational workflow to characterize the structural and binding differences between transketolase (TKT) and transketolase-like 1 (TKTL-1) and to identify selective inhibitory scaffolds. In the absence of an experimental TKTL-1 crystal structure, a multi-template homology modeling approach using high-identity (>57%) and high-coverage (>96%) templates produced a robust 3D model. Structural validation through Ramachandran analysis (>98% favored/allowed residues), residue energy distribution, and pLDDT scores confirmed the model’s reliability for structure-based design. Local quality assessments further demonstrated high confidence (>90%) for most residues, especially within the binding site, with only minor reductions at the terminal regions, common in homology models and unlikely to affect core binding site predictions.

Docking protocol validation with TKT (PDB ID: 4KXW) accurately reproduced the crystallographic TDP-binding pose (RMSD < 0.3 Å) and recapitulated key interactions with His103, Asp483, and Asn187, consistent with previous reports [40]. This confirmed the robustness of our computational approach. Comparative docking and MM-PBSA analysis revealed a pronounced reduction in TDP affinity for TKTL-1 (−6.9 kcal/mol; ΔG_bind_ = +11.43 kJ/mol) compared to TKT (−7.8 kcal/mol; ΔG_bind_ = −364.16 kJ/mol). The weakened affinity stems from the absence of Asp483 and a 38-residue deletion in TKTL-1, which disrupt cofactor coordination. These structural differences provide a clear mechanistic basis for its thermodynamically unfavorable binding and support its classification as a non-catalytic, regulatory variant. The lack of these critical residues highlights TKTL-1’s distinct biochemical profile (Figure 7), aligning with prior studies on the divergent functional roles of TKT and TKTL-1 [23,29]. Sequence alignment confirms that this deletion removes essential TDP-binding residues, including His103, while retaining enough structural homology to support protein–protein interactions [29,30,31]. Thermodynamic results offer compelling evidence that TKTL-1 cannot form a stable TDP complex under physiological conditions, resolving earlier debates on its enzymatic activity [21,22].

To explore therapeutic implications of this reduced TDP affinity, we performed binding site mapping using CASTp [41], receptor cavity analysis in Discovery Studio [38], and blind docking (Figure 15). All three methods consistently identified a druggable cavity of 485 Å^3^ with a favorable draggability score of 0.87. The site comprised 40 key residues, including Ala436, Asn437, Lys439, and Gly440—terminal residues critical for TKTL-1’s interaction with TKT monomers [29]. This presents a unique targeting opportunity, as these residues are not present in the same configuration in TKT, enabling the possibility of selective TKTL-1 inhibition without affecting normal TKT function. Based on these findings, we developed a pharmacophore model incorporating six essential features: hydrogen bond donors, hydrogen bond acceptors, aromatic centers, hydrophobic regions, and electrostatic interaction sites with defined spatial constraints (Figure 16). This model provides a rational framework for identifying and optimizing TKTL-1-selective modulators. Guided by these parameters, we proposed three heterocyclic scaffold classes with distinct mechanisms for targeting TKTL-1: quinaolzine-, benzimidazole- and pyrimidine- based derivatives. These scaffolds were generated based on the pharmacophore features and structural pocket analysis.

Collectively, these results provide definitive computational evidence that TKTL-1 lacks enzymatic TDP-binding capacity, clarify its probable regulatory role in cancer metabolism, and establish the first comprehensive structure-based drug design framework for this target. The identification of a unique allosteric site and highly selective scaffold chemotypes offers promising starting points for experimental validation and therapeutic development.

This work advances previous computational studies by delivering rigorous thermodynamic validation of binding differences and introducing targeted inhibitor design. The integration of multiple validation strategies—redocking verification, multi-template homology modeling, and convergent binding site identification, provides exceptional confidence in our predictions. Furthermore, the discovery of druggable sites with high selectivity potential marks a novel contribution absent in previous reports [28], positioning this study as the first to comprehensively define TKTL-1 as a structure-based drug design target.

Several limitations of this work should also be acknowledged. The absence of molecular dynamics simulations restricts our ability to capture dynamic binding behaviors, induced-fit effects, and potential allosteric communication pathways, while the use of static homology models, despite rigorous validation, may not fully represent all biologically relevant conformational states. Simplified solvation models and the omission of entropic contributions in some binding energy calculations may influence the absolute accuracy of predicted affinities, although the comparative selectivity trends between TKT and TKTL-1 are likely to remain reliable. Most importantly, the complete lack of experimental validation means that all results remain predictive and require empirical confirmation. Addressing these limitations will be a priority in future work, beginning with molecular dynamics simulations using explicit solvent models to explore binding dynamics and allosteric effects, followed by experimental studies involving TKTL-1 protein expression, biophysical binding assays such as surface plasmon resonance (SPR) and isothermal titration calorimetry (ITC), and cell-based functional assays. Ultimately, the synthesis and evaluation of the proposed lead compounds (QZ-TKTL-001, BZ-TKTL-003, and PY-TKTL-007) will be essential to validate the computational predictions and advance these scaffolds toward therapeutic application.

The overexpression of TKTL-1 in multiple cancer types [20,23,24,25,26,27,42], together with its regulatory role in nucleotide synthesis pathways [29], underscores its potential as a promising target for metabolic cancer therapy. The computational framework developed in this study lays the groundwork for structure-based optimization of TKTL-1 modulators and represents a significant step toward translating these findings into clinical applications. Furthermore, the reported link between TKTL-1 and the tumor suppressor gene CDH-1 via the D-box sequence [33] offers additional therapeutic avenues, including the possibility of combination strategies that inhibit TKTL-1 activity while restoring normal cell cycle control [29]. Beyond oncology, TKTL-1 modulation may have relevance in neurodegenerative diseases and diabetic complications [21,37], where altered TKTL-1 function has been implicated. The high selectivity of the designed compounds described here could enable targeted intervention in these diverse pathological contexts while minimizing metabolic disruption in healthy tissues.

## 4. Materials and Methods

### 4.1. Computational Methodology Overview

All computational studies were performed using Discovery Studio 2020 Client v20.1 (BIOVIA, Dassault Systèmes, San Diego, CA, USA) [38] and Gaussian 09 (Revision D.01) [43]. The human TKTL-1 sequence (UniProt ID: P51854, 596 residues) was used for all modeling studies.

### 4.2. Multi-Template Homology Modeling

Five high-resolution crystal structures were selected as templates based on sequence identity (>57%) and structural coverage (>96%): 4KXW (63% identity), 6HAD (61% identity), 6RJB2 (61% identity), and 3OOY (57% identity). Homology models were generated using MODELLER 9.24 with loop optimization and energy minimization [44]. Model quality was assessed using Ramachandran plot analysis (RAMPAGE) and Z-score evaluation. The 4KXW-based model showed the highest quality (97.7% residues in favored regions, Z-score = −0.425) and was selected for subsequent studies.

### 4.3. Molecular Docking and Binding Analysis

#### 4.3.1. Protocol Validation

Redocking of thiamin diphosphate (TDP) to human TKT (PDB ID: 4KXW) was performed to validate computational protocols. The redocked pose showed excellent agreement with crystal structure (RMSD < 0.3 Å) and literature binding energy values (−7.8 kcal/mol).

#### 4.3.2. TKTL-1 Docking Studies

TDP was docked to the TKTL-1 homology model using LibDock and CDOCKER algorithms. Ligand preparation included geometry optimization (MMFF94 force field) and conformational sampling. Protein preparation involved hydrogen addition, charge assignment, and energy minimization. Binding energies were calculated and converted to dissociation constants using standard thermodynamic relationships.

### 4.4. Binding Site Identification and Characterization

Three independent methods were employed: (1) CASTp cavity analysis for volume and draggability assessment, (2) Discovery Studio receptor cavity identification for site scoring, and (3) blind docking for binding hotspot validation. Surface properties including hydrophobicity, electrostatic potential, and hydrogen bonding capability were analyzed using molecular surface algorithms.

### 4.5. Quantum Chemical Optimization

Individual molecules and complexes were optimized using Gaussian 09 with RHF/6-31+G basis set (UHF for open-shell systems). Full geometry optimization was performed with frequency validation to confirm energy minima.

### 4.6. Statistical Analysis and Validation

All calculations were performed in triplicate with statistical analysis using standard deviation. Interaction networks were analyzed using distance and angle criteria for hydrogen bonds (<3.5 Å, >120°), electrostatic interactions (<5 Å), and hydrophobic contacts (<4.0 Å). Results were validated against experimental literature values where available.

## 5. Conclusions

In conclusion, this comprehensive computational study provides strong evidence for the distinct functional role of TKTL-1 and establishes a robust framework for its therapeutic targeting. By integrating high-quality structural modeling, detailed thermodynamic analysis, and rational drug design, we deliver new insights into TKTL-1 biology and identify opportunities for selective metabolic intervention in cancer. The striking difference in binding free energy between TKT and TKTL-1 for TDP, coupled with the discovery of unique druggable pockets and the proposal of novel heterocyclic scaffolds, offers both mechanistic clarity and practical starting points for therapeutic development. While docking and selectivity results remain to be validated, the scaffold designs presented here set the stage for future computational and experimental investigations.

These findings not only advance our understanding of TKTL-1’s contribution to cancer metabolism but also highlight its broader therapeutic potential in conditions where altered TKTL-1 activity is implicated. Future work will focus on validating these predictions through protein expression, biophysical assays, and cell-based functional studies, as well as exploring the pharmacokinetics, safety, and efficacy of the lead compounds in preclinical models. Such efforts will be essential to translate the computational advances presented here into clinically relevant strategies for targeting TKTL-1 in cancer and other diseases.

## Figures and Tables

**Table 1:** Five templates used for homology modeling generation.

Template (PDB ID)	Align Score	Cover (%)	Resolution (Å)	Z Score
4KXW	1896	98	0.97	−0.425
6HAD	1893	97	1.04	−0.519
6RJB~1	1890	98	1.15	−0.548
6RJB~2	1896	96	1.15	−0.687
3OOY	1885	96	2.05	−0.564

**Table 2:** The number of residues in the favored, allowed, and outlier regions.

Template (PDB ID)	No. of Residues in Favored Region	No. of Residues in Allowed Region	No. of Residues in Outlier Region
4KXW	1153 (97.7%)	25 (2.1%)	2 (0.2%)
6HAD	1142 (96.8%)	36 (3.1%)	2 (0.2%)
6RJB~1	1151 (97.5%)	27 (2.3%)	2 (0.2%)
6RJB~2	1151 (97.5%)	28 (2.4%)	1 (0.1%)
3OOY	1143 (97.2%)	31 (2.6%)	2 (0.2%)

**Table 3:** Thiamin diphosphate optimization.

Field	Value
File Type	.chk
Calculation Type	FOPT
Calculation method	RHF
Basis set	6-31+G
Charge	−2 (physiological pH)
Spin	singlet
Solvation	PCM (water)
SCF Convergence	8.0 × 10^−6^ Hartee/Bohr
Imaginary Freq	0 (minimum)
Dipole Moment	12.456 Debye
Point Group	C1

**Table 4:** Transketolase (TKT) optimization.

Field	Value
File type	.chk
Calculation type	FOPT
Calculation method	UHF
Basis set	6-31+G
Charge	0
Spin	Doublet
Solvation	Gas phase
SCF convergence	6.0 × 10^−6^ Hartree/Bohr
Imaginary Freq	0 (minimum)
Dipole moment	6.58 Debye
Point group	C1

**Table 5:** Transketolase-like 1 (TKTL-1) optimization.

Field	Value
Field Type	.chk
Calculation type	FOPT
Calculation method	RHF
Basis set	6-31+G
Charge	0
Spin	Singlet
Solvation	Gas Phase
SCF Convergence	5.0 × 10^−6^ Hartree/Bohr
Imaginary Freq	0 (minimum)
Dipole Moment	7.731 Debye
Point Group	C1

**Table 6:** Transketolase (TKT) and Thiamin diphosphate (TDP) complex optimization.

Field	Value
File Type	.chk
Calculation type	FOPT
Calculation method	UHF
Basis set	6-31+G
Charge	0
Spin	Doublet
Solvation	Gas Phase
SCF Convergence	1.3 × 10^−2^ Hartree/Bohr
Imaginary Freq	0 (Minimum)
Dipole Moment	4.816 Debye
Point group	C1

**Table 7:** Transketolase-like 1 (TKTL-1) and Thiamin Diphosphate (TDP) complex optimization.

Field	Value
File Type	.chk
Calculation type	FOPT
Calculation Method	RHF
Basis Set	6-31+G
Charge	0
Spin	Singlet
Solvation	Gas Phase
SCF Convergence	1.0 × 10^−6^ Hartree/Bohr
Imaginary Freq	0 (Minimum)
Dipole Moment	8.44 Debye
Point group	C1

**Table 8:** Summary of geometry optimization results for TDP, TKTL-1, TKT, and complexes.

System	Method	Spin State	Solvation	Dipole Moment (Debye)	Stability Assessment
Thiamine diphosphate (TDP)	RHF/6-31+G	Singlet	PCM (water)	12.456	Stable, highly polar; true minimum
TKTL-1	RHF/6-31+G	Singlet	Gas phase	7.731	Stable; strong polarity; regulatory protein candidate
TKT	UHF/6-31+G	Doublet	Gas phase	6.580	Stable minimum; moderate polarity
TKTL-1–TDP complex	RHF/6-31+G	Singlet	Gas phase	8.449	Stable; polar interface; weak binding
TKT–TDP complex	UHF/6-31+G	Doublet	Gas phase	4.816	Stable; moderate polarity; strong binding

## Data Availability

The authors declare that the data supporting the findings of this study are addressed within the article.
